# O-21 - COPRODUCING AND EVALUATING PUBLIC HEALTH COMMUNICATION FOR CLOSE CONTACTS OF INVASIVE GROUP A STREPTOCOCCAL INFECTION (IGAS) CASES WHO ARE EXPERIENCING HOMELESSNESS AND OR INJECT DRUGS

**DOI:** 10.1093/pubmed/fdag046.018

**Published:** 2026-07-09

**Authors:** Dr Alex Martin, Dr Sarah Denford, Nicky Wilsenham, Libby Eastwood, Professor Lucy Yardley, Professor James Rubin, Dr Theresa Lamagni, Professor Richard Amlot, Dr David Roberts

**Affiliations:** King's College London; University of Bristol; The Society of St. James; UK Health Security Agency, Chief Scientific Officer’s Group; University of Bristol; King's College London; UK Health Security Agency, Antimicrobial Resistance & Healthcare-Associated Infection Division; UK Health Security Agency, Chief Scientific Officer’s Group; UK Health Security Agency South East

## Abstract

**Background:**

Invasive group A streptococcal (iGAS) disease is a rare but serious bacterial infection affecting a broad cross-section of people. People experiencing homelessness (PEH) and people who inject drugs (PWID) are disproportionately affected. Public health ‘warn and inform’ advice is routinely provided to close contacts of iGAS cases to encourage early healthcare presentation given their elevated risk of disease. Risk communication materials may not be accessible, understandable, or actionable for PWID and PEH.

**Objectives:**

This project (1) explored the experiences and communication needs of PEH and PWID; (2) coproduced iGAS factsheets with PEH and PWID; and (3) evaluated whether the coproduced materials were accessible, understandable, non-stigmatising, and actionable.

**Methods:**

We used the Agile Co-production and Evaluation (ACE) framework to guide coproduction and mixed-methods evaluation through in-person group conversations and a survey. Together with support service staff and people with lived experience, we developed guiding principles and iteratively optimised and evaluated iGAS public health materials. A multidisciplinary steering group including expertise in health protection, epidemiology, behavioural science, support service provision, and research guided the study.

**Results:**

Coproduction activities informed factsheet modifications incorporating simplified language, visual cues, and relevant content. Coproducers described the factsheet as eye-catching, easy to read, and person-centred whereas the original version was viewed as having limited relevance to their needs. In the evaluation survey, 32/39 responders preferred the coproduced factsheet, which was rated higher for readability and clarity of guidance. Symptoms of invasive group A streptococcal infection were correctly identified and responders stated that they would follow the advice.

**Conclusions:**

Our findings show the potential of rapid, participatory approaches to improve and evaluate the design of public health advice for underserved groups. By applying the ACE framework, we showed that coproduced materials were clearer, more actionable, and better aligned with the needs of PEH and PWID.

